# Effects of social confinement during the first wave of COVID-19 in Mexico City

**DOI:** 10.3389/fpubh.2023.1202202

**Published:** 2023-06-23

**Authors:** Stephany Segura-García, Ameyalli Barrera-Ramírez, Guadalupe O. Gutiérrez-Esparza, Elizabeth Groves-Miralrio, Mireya Martínez-García, Enrique Hernández-Lemus

**Affiliations:** ^1^Health Promotion Program, Universidad Autónoma de la Ciudad de México, Mexico City, Mexico; ^2^Cátedras CONACYT, Consejo Nacional de Ciencia y Tecnología, Mexico City, Mexico; ^3^Department of Immunology, National Institute of Cardiology Ignacio Chávez, Mexico City, Mexico; ^4^Computational Genomics Division, National Institute of Genomic Medicine, Mexico City, Mexico; ^5^Center for Complexity Sciences, Universidad Nacional Autónoma de México, Mexico City, Mexico

**Keywords:** social confinement, COVID pandemic, mental health, domestic violence, sleep disturbances, feeding habits, social support, anxiety

## Abstract

**Background:**

The COVID-19 pandemic led to global social confinement that had a significant impact on people's lives. This includes changes such as increased loneliness and isolation, changes in sleep patterns and social habits, increased substance use and domestic violence, and decreased physical activities. In some cases, it has increased mental health problems, such as anxiety, depression, and post-traumatic stress disorder.

**Objective:**

The objective of this study is to analyze the living conditions that arose during social confinement in the first wave of COVID-19 within a group of volunteers in Mexico City.

**Methods:**

This is a descriptive and cross-sectional analysis of the experiences of volunteers during social confinement from 20 March 2020 to 20 December 2020. The study analyzes the impact of confinement on family life, work, mental health, physical activity, social life, and domestic violence. A maximum likelihood generalized linear model is used to determine the association between domestic violence and demographic and health-related factors.

**Results:**

The findings indicate that social confinement had a significant impact on the participants, resulting in difficulties within families and vulnerable conditions for individuals. Gender and social level differences were observed in work and mental health. Physical activity and social life were also modified. We found that suffering from domestic violence was significantly associated with being unmarried (*OR = 1.4454, p-value = 0.0479*), lack of self-care in feeding habits (*OR = 2.3159, p-value = 0.0084*), and most notably, having suffered from a symptomatic COVID-19 infection (*OR = 4.0099, p-value = 0.0009*). Despite public policy to support vulnerable populations during confinement, only a small proportion of the studied population reported benefiting from it, suggesting areas for improvement in policy.

**Conclusion:**

The findings of this study suggest that social confinement during the COVID-19 pandemic had a significant impact on the living conditions of people in Mexico City. Modified circumstances on families and individuals, included increased domestic violence. The results can inform policy decisions to improve the living conditions of vulnerable populations during times of social confinement.

## 1. Introduction

At the end of 2019, the SARS-CoV-2 virus, which causes the coronavirus disease 2019 (COVID-19), was identified, which since then has rapidly spread throughout the world. Social confinement or isolation was the most important public health measure adopted by most countries to mitigate, attend to, and control the spread, as well as the effects of the pandemic during the first wave of COVID-19 (1). Mexico was no exception, with ~126 million inhabitants, and social confinement was based on the suspension of non-essential activities or those that would not affect the substantive activities of a public, social, or private organization such as activities in schools, offices, public works, factories, and/or some services (2). This measure was part of the so-called *National Season of Healthy Distance* (Jornada Nacional de Sana Distancia, in Spanish), a mandatory-yet-not legally reinforced social confinement strategy that started on 23 March 2020, postponed until 30 May 2020, and was accompanied by a *modulated reopening strategy* by an epidemiological traffic light starting 1 June 2020, which established the measures of social de-confinement depending on the spread of the virus in different regions of the country (3, 4). At the end of December 2020, the official data for Mexico City (CDMX), the national epicenter of the pandemic, reported around 264,000 confirmed cases of infected people (5).

More than 3 years after the start of the pandemic, we know that social confinement resulted in the partial or total cancellation of many formal or informal work activities, with strong impacts on the economy and severe consequences for the daily life routine of many families (6–10). Around the world, unemployment figures rose rapidly to double digits, with millions of people signing up for welfare payments, being women more affected than men by the economic instability (11, 12). The highest unemployment rates reported in Mexico in 2020 were located in the months of June, July, and August with an average of 2.8 million unemployed, while informal employment went from 20.7 million in April to 28.1 million in September, with a continuous increase during the following months (13). A study reported that during the same period, Mexican women were the ones most affected by unemployment and most of them have not yet recovered from it (14).

To date, various studies have explored living conditions in the context of lockdown and social distancing from an academic perspective in order to understand the aftermath that isolation has left on society (15–19). In general, we know that changes in the routine of lifestyle and the lack of physical contact with friends and family negatively affect the mental health of people of all ages (20). During the first months of the COVID-19 pandemic, stress, frustration, depression, anxiety, and panic disorder became integral parts of adult life. The presence of chronic illnesses, fear of acquiring the infection, the angst of infecting or losing a loved one, or the uncertainty of not having enough resources to survive have disrupted the dynamics of many families (21).

The social isolation and stay-at-home policies also contributed to increased vulnerabilities related to mental health, including domestic violence, which may manifest in physical, psychological, or economic forms (7, 22). In Mexico City, as in many regions of Latin America, confinement exacerbated this social phenomenon that has plagued society for decades, the domestic violence and the deterioration in mental health conditions, often related to economic recession, poverty, unemployment, school dropout, addiction, housing crisis, and reduced options for support, among other factors (23–25). The purpose of this article is to analyze the living conditions that occurred or were modified during the first wave of COVID-19 social confinement in a cohort from a metropolitan population in Mexico City. Our focus was set on exploring the presence of particular diseases, the modification of daily-life habits, the experiencing of episodes of violence, and the social support received as part of the follow-up of a group of volunteers participating in a cohort of CDMX.

## 2. Methods

### 2.1. Information retrieval

This research was conducted during the COVID-19 health emergency, thus all *fieldworks* were conducted online. The design chosen was an online self-report questionnaire with 24 questions applied to follow-up volunteer adults from Mexico City. Based on online platforms and/or email, the data collection approach has not only proven to be a cost-effective survey alternative for collecting large amount of data in a short period of time but it also appears to be an effective strategy for collecting data on sensitive topics among vulnerable populations (26). The questionnaire was sent via email and WhatsApp messages. The invitation to participate in the study was sent up to three times in some cases as a reminder and/or to give the volunteers more time to send their responses. The initial message explained the objective of the study, the confidentiality of the replies, and stated that the information would be used only for research purposes. At the end of the study, an acknowledgment letter was sent to the volunteers to thank them for their participation. One of the major goals of the survey was to evaluate the social vulnerability of some CDMX families during the COVID-19 pandemic.

For the purposes of this research, 12 of the 24 questions were selected. The form included questions to know the general health conditions, violet situations, and the individual perspective on the impact of the COVID-19 pandemic (see Tables 1, 2). The answer options could be multiple choice or open-ended. Some answers were classified for their systematization and subsequent analysis from pre-established codes. In the case of the variable *type of violence*, the answers were coded as: economic, psychological, verbal, emotional, symbolic, physical, or unspecified, according to the literature reviewed (27). The health conditions were classified according to the International Classification of Diseases, 10th Revision (ICD-10) (28). Demographic information such as sex, age, marital status, and level of social development were also recorded. The Mexican Social Development Index (SDI) classifies population development from worse (less development) to best (more social development) into four levels as follows: (1) very low, (2) low, (3) medium, and (4) high (29).

**Table 1:** Self-report formulaire (sociodemographic, comorbidities, and habits modified).

**Features**	**Abbreviation**	**Possible answers**
Sex	Sex	Man
		Woman
What is your current marital status?	Marital status	Married
		Unmarried
What has been your main workplace during the pandemic?	Work	Business office
		Health services
		Merchant
		Home
		Outdoor work
		Unemployed
Have you been diagnosed with COVID-19?	COVID-19 diagnose	Yes, with lab tests
		Yes, with symptoms
		No
In case your previous response was affirmative, you were at:	Place of treatment	Hospital
		Home
Do you have any of the following diseases?	Ailments	Cardiovascular disease
		Respiratory disease
		Diabetes
		Arterial hypertension
		Obesity
		Metabolic syndrome
		Alcoholism
		Smoking
		None
During the lockdown, What habits have you changed?	Modified habits	Feeding
		Sleeping
		Physical activity
		Social life
		None

**Table 2:** Self-report formulaire (lifestyle characteristics, violence episodes, and support received).

**Features**	**Abbreviation**	**Possible answers**
Do you exercise at home		Yes
		No
How often did you exercise at home?	Low-impact	Yes, at least every third day, for half an hour
	Moderate	Yes, every third day, for more than an hour or
		Yes, more than three times a week at least half an hour
	High-impact	Yes, more than three times a week for more than an hour
Have you taken care of your feeding habits	Feeding	Yes
		Sometimes
		A few times
		No
Do you consider that since lockdown for COVID-19, you have experienced situations of family violence in your home?	Violence	Yes
		No
How often have been this violence situations?	Frequency of violence	Sometimes
		Several times
		Many times
In the affirmative case, could you briefly describe what kind of violent situations have you experienced at home?	Type of violence	Description
Do you receive some kind of support?	Support received	Financial
		Social
		Food
		Psychological
		Medical
		None

### 2.2. Statistical analysis

The data analysis was carried out with R/Rstudio version 4.0.2. A descriptive analysis of the general characteristics of the studied population was carried out. The chi-square test was used to check for differences between men and women. Statistical significance was determined at *p*_*value*_ < 0.05. A multivariate logistic regression model was fitted to estimate the association between *Violence* and independent variables (age, sex, marital status, social stratum, COVID-19 diagnosis, work during the pandemic, and some habits such as feeding, sleeping, or physical activity) in the form of a generalized linear model with a binomial link function. Model optimization (stepwise regression) was performed using maximum likelihood calculations to choose the best model compatible with the data. The maximum likelihood criterion in the likelihood ratio test was Wilk's test. Variance inflation factor (VIF) determination was calculated for each regression model to assess for multi-collinearity. *VIF* < < 10 for all retained variables. Odds ratios (ORs) and 95% confidence intervals (2.5–97.5%C.I.) were calculated to estimate the strength of the association. All tests were performed at a confidence level of α = 0.05. The balance between sensitivity and specificity was evaluated using ROC curves and calculation of the area under the ROC curve (AUROC).

## 3. Results

### 3.1. General features

Out of the 2,440 forms sent, 1,629 responses were obtained and included in the analysis after meeting the predefined selection criteria (consent to participate in the study, non-duplicate records and complete data, responses received within the period of the first wave of COVID-19 infections). In total, 34% of the volunteers were men, with a median age of 41 years (IQR 33–48) and 66% were women, with a median age of 42 years (IQR 33–49). The percentage of respondents who were married was higher among men than women (55.76 vs. 48.64%, respectively, < 0.0001).

During the first wave of infections, 3.31% of participants had a laboratory-confirmed COVID-19 diagnosis, and 2.15% had the suspicion of having suffered from it based on the presented symptoms. Home was the main place of care and/or treatment (95.51%). Of the cases diagnosed via a laboratory test, men reported slightly more infections than women, 3.24 and 3.36%, respectively (see Table 3). Regarding the employment situation during confinement, the condition of unemployment was reported more by women (15.94%) than by men (8.81%); for those who kept their jobs, it was mainly carried out from home office (55.58 and 59.18%, respectively, *p*_*value*_ = 0.0011), followed by business office (15.11 and 9.69%, respectively, *p*_*value*_ = 0.0087) and outdoor work (7.73 and 1.68%, respectively, *p*_*value*_ = 0.0056).

**Table 3:** Distribution of sociodemographic, comorbidities, and habits modified during the first wave of COVID-19 social confinement.

**Features**	**Total 1,629**	**Man 556 (34%)**	**Woman 1,073 (66%)**	***p*-value**
**Marital status** ^*^				
Married	51.07	55.76	48.65	<**0.0001**
Unmarried	48.93	44.24	51.35	**0.0135**
**Social stratum** ^*^				
1) Very low	11.48	11.69	11.37	0.1420
2) Low	36.89	36.15	37.28	**0.0077**
3) Medium	25.48	25.72	25.35	**0.0195**
4) High	26.15	26.44	26.00	**0.0173**
**Work** ^*^				
Business office	11.54	15.11	9.69	**0.0087**
Health services	9.15	7.91	9.79	0.4903
Merchant	4.11	4.86	3.73	0.3028
Home office	57.95	55.58	59.18	**0.0011**
Outdoor work	3.74	7.73	1.68	**0.0056**
Unemployed	13.51	8.81	15.94	0.9805
**COVID-19** ^*^				
With laboratory tests	3.31	3.24	3.36	0.6885
With symptoms	2.15	2.70	1.86	0.5244
No	94.54	94.06	94.78	<**0.0001**
**COVID-19 place of treatment** ^*, **^				
Home	95.51	94.87	96.00	<**0.0001**
Hospital	4.49	5.13	4.00	0.2893
**Presence of some diseases** ^*^				
Certain infectious and parasitic diseases	0.37	0.18	0.47	0.9999
Diseases of the circulatory system	4.41	3.24	5.03	0.9999
Diseases of the digestive system	2.02	1.26	2.42	0.9999
Diseases of the genitourinary system	0.74	0.36	0.93	0.9999
Diseases of the respiratory system	3.68	3.42	3.82	0.7244
Diseases of the nervous system	0.37	0.54	0.28	0.9999
Diseases of the musculoskeletal system	1.17	0.90	1.30	0.9999
Endocrine, nutritional, and metabolic diseases	22.59	22.84	22.46	**0.0288**
Neoplasms	0.31	0.00	0.47	0.9999
Mental and behavioral disorders	10.13	12.77	8.76	**0.0243**
**Modified** ^*^				
Feeding	15.22	13.49	16.12	0.2593
Sleeping	17.19	16.19	17.71	0.1316
Physical activity	27.01	26.98	27.03	**0.0189**
Social life	35.48	38.13	34.11	**0.0009**
None	5.10	5.22	5.03	0.4195

^*^Values expressed in percentage.

^**^Values calculated only for cases of self-reported COVID-19 (54 men and 35 women).

The bold values indicate the statistically significant results.

### 3.2. Presence of some diseases

The *main diseases*, as reported by the participants, broadly belong to the following classes: (1) *Endocrine, Nutritional, and Metabolic Diseases (22.59%)*, e.g., those related to diabetes mellitus, obesity, and metabolic syndrome (ICD-10: E00-E90); (2) *Mental, Behavioral, and Neurodevelopmental Disorders (10.13%)*, which include alcoholism, smoking, and anxiety and depression (ICD-10: F00-F99), among others; (3) *Diseases of the Circulatory System (4.41%)*, including hypertension, cardiovascular disease, venous insufficiency, and also some arrhythmias (ICD-10: I00-I99); and (4) *Diseases of the Respiratory System (3.68%)*, such as infectious and chronic respiratory diseases, allergic rhinitis, asthma, sinusitis, and chronic bronchitis (ICD-10: J00–J99).

We can observe that men are more frequently affected by endocrine, nutritional, and metabolic diseases than women (22.84 and 22.46%, respectively). Similarly, mental and behavioral disorders were more prevalent among women (12.77%) than men (10.13%), while the opposite trend was found in the case of circulatory diseases (5.03% in women and 3.24% in men) and respiratory system-related diseases (3.82% in women and 3.42% in men). Statistically significant differences were found between men and women in the categories of endocrine, nutritional, and metabolic diseases (*p*_*value*_ = 0.0288), as well as mental and behavioral disorders (*p*_*value*_ = 0.0243; see Table 3). Furthermore, it is worth noting that only women reported suffering from neoplasms such as cervical, colon, and breast cancer (0.47%; ICD-10, C00–D48), albeit in smaller proportions.

### 3.3. Modified habits

Among the *self-reported habits that were modified* during social confinement between men and women, substantive changes in social life were indicated (38.13 and 34.11%, respectively, *p*_*value*_ = 0.0009), as was to be expected due to confinement, followed by physical activity (26.98% in men and 27.03% in women, *p*_*value*_ = 0.0189). We could notice that habits related to feeding and sleeping changed to a lesser extent in men than in women, without being statistically significant (see Table 3).

Regarding exercise at home, a higher percentage was reported in men than in women in low impact—*at least every third day for half an hour* (25 and 24.88%, respectively, *p*_*value*_ = 0.0234), for moderate impact (*every third day, for more than an hour or more than three times a week at least half an hour*) 25.18% in men and 19.66% in women (*p*_*value*_ = 0.0028), and for high impact—*more than three times a week for more than 1 h*, 6.53 and 4.55% were reported between men and women, respectively (*p*_*value*_ = 0.2335). Regarding nutrition care, the majority of the participants reported taking care of it, both men and women (45.50 and 44.64%, respectively); among them, also a statistically significant difference was found (*p*_*value*_ = 0.0012; see Table 4).

**Table 4:** Distribution of lifestyle characteristics, violence episodes, and support received during the first wave of COVID-19 social confinement.

**Features**	**Total (1,629)**	**Man (556)**	**Woman (1,073)**	***p*-value**
**Exercise at home** ^*^				
Low-impact	24.92	25.00	24.88	**0.0234**
Moderate-impact	21.55	25.18	19.66	**0.0028**
High-impact	12.28	11.51	12.67	0.2335
No	41.25	38.31	42.78	**0.0149**
**Feeding habits care** ^*^				
Yes	44.94	45.50	44.64	**0.0012**
Sometimes	38.06	35.97	39.14	**0.0143**
A few times	11.97	13.67	11.09	**0.0439**
No	5.08	4.86	5.13	0.5247
**Violence** ^*^				
Yes	9.09	8.27	9.51	0.3922
**Frequency of violence** ^*, **^				
Sometimes	6.88	6.29	7.18	0.2412
Many times	0.37	0.36	0.37	0.9999
Several times	1.84	1.62	1.96	0.9288
**Type of violence** ^*, **^				
Economic	8.11	13.04	5.88	**0.0001**
Emotional	28.38	21.74	31.37	<**0.0001**
Physical	8.78	6.52	9.80	0.4662
Not specified	4.73	8.69	2.94	**0.0012**
Psychological	50.68	41.30	54.90	<**0.0001**
Symbolic	4.05	0.00	5.88	0.2603
Verbal	33.78	28.26	36.27	**0.0009**
**Support received** ^*^				
Financial	9.76	10.61	9.31	0.1154
Social	5.65	5.03	5.96	0.6190
Food	2.58	1.61	3.07	0.9999
Psychological	6.14	4.86	6.80	0.8136
Medical	8.83	7.91	9.31	0.4336

^*^Values expressed in percentage.

^**^Values calculated only for cases of self-reported violence (46 men and 102 women).

The bold values indicate the statistically significant results.

### 3.4. Violence episodes

During the first period of confinement, ~9% of the volunteers self-reported having experienced *some forms of violence* within their home. Episodes of violence were registered less often in men than in women (8.27 and 9.51%, respectively, *p*_*value*_ = 0.3922). The main types of violence were related to the partner, parents, children, or other relatives and were coded as psychological violence (50.68%), emotionals (28.38%), and verbal (33.78%). The frequency with which violence occurred in the home between men and women was described as follows: sometimes (6.29 and 7.18%, respectively, *p*_*value*_ = 0.2412), several times (1.62 and 1.96%, respectively, *p*_*value*_ = 0.9288) and in smaller proportions, and many times (0.36 and 0.37%, respectively). Statistically significant differences were found between men and women in the types of violence: psychological (41.30 and 54.90%, respectively, < 0.0001), verbal (28.26 and 36.27%, respectively, *p*_*value*_ = 0.0009), and economic (13.04 and 5.88%, respectively, *p*_*value*_ = 0.0001; see Table 4).

Some open-ended responses from participants who experienced violence described the type of violence. Examples include: “*My partner used to mock my crying (my mother died of COVID on May 2020) and he was not patient with my 3-year-old son who had many tantrums, often he kicked us out of his mother's house where I was spending my isolation, ... he told me many times that I shouldn't continue traumatized..*.”; “*My sister-in-law threatened to hit me with the aid of her whole family*”; “*Death threats, insults, and hits*.” In the same way, indirect or systemic types of violence were identified, such as: “*Emotional violence by my brother-in-law, since my sister lives in my parents' house and he hits her and my nephew and that affects me in some way, because I see it almost every day. The man is an alcoholic*.”; “*We are three people living together, we have economic and social problems, we have a small business that is in danger of disappearing due to the contingency, and also it is a little difficult not to argue while being inside the house*.”; “*I have suffered from Machismo related to domestic tasks from my partner*.”

### 3.5. Support received

As for “support received”, the majority of the participants (69%) indicated that they did not receive any. However, of those who had some support, women reported receiving more medical (9.31 vs. 7.91%), psychological (6.80 vs. 4.86%), social (5.96 vs. 5.03%), and food support than men (3.07 vs. 1.61%, respectively), while men indicated receiving only economic support more than women (10.61 and 9.31%, respectively; see Table 4).

### 3.6. Multivariate logistic regression model

Regarding the logistic regression model, the occurrence of violence (as the dependent variable) was mainly associated with age (*p*_*value*_ = 0.0060), being unmarried (*p*_*value*_ = 0.0479), not having taken care of their feeding habits (*p*_*value*_ = 0.0084) and with the self-reported variable of having presented symptoms of COVID-19 (*p*_*value*_ = 0.0009). Older participants had a slightly yet significantly higher risk of experiencing episodes of violence than younger ones (OR = 1.02). Similarly, those participants who worked at home during confinement or who did not have a job and remained in a shelter had higher risks (OR = 1.05 and OR = 1.75, respectively) of suffering a violent event, in contrast to those whose work was carried out outside the home, such as in sectors such as commerce, health services, or in an office (OR = 0.39, OR = 0.87, OR = 0.70) who experienced a lower probability of experiencing violence at home.

The participants who did not perform any exercise at home (OR = 1.24) or who exercised with low-impact activity (OR = 1.49) were found also to have more probability of experiencing episodes of violence than those who performed moderate-impact exercise (OR = 0.71). A similar pattern was observed for participants who did not take care of their diet or did not take enough care of it (OR = 2.31 and OR = 1.13). Moreover, those volunteers who reported having COVID-19 either with symptoms or with a laboratory test were also at greater risk of suffering episodes of violence at home, unlike those who had not experienced this condition. The AUROC for our overall model was 0.6698405, a value indicating a relatively good model performance for this type of study (30). The full maximum likelihood, adjusted odds ratios (95% CI), and *p*-values of the final (maximum likelihood) model are presented in Table 5.

**Table 5:** Logistic regression model, considering *Violence* as the outcome variable.

**Variables**	**Estimate**	**[2.5% C.I.]**	**[97.5% C.I.]**	**OR**	***p*-value**
Intercept	−3.81763	−5.252191533	−2.47659362	0.0220	<**0.0001**
Age	0.02880	0.008393227	0.04952561	1.0292	**0.0060**
Unmarried	0.36835	0.003894635	0.73486521	1.4454	**0.0479**
Work^§^					
(Home office)	0.05688	−0.740232917	0.99661633	1.0585	0.8965
Work^§^					
(Merchant)	−0.92503	−2.517180094	0.43539035	0.3965	0.2038
Work^§^					
(Unemployed)	0.56231	−0.298563685	1.54541383	1.7547	0.2262
Work^§^					
(Health services)	−0.12805	−1.128519539	0.94089512	0.8798	0.8058
Work^§^					
(Business office)	−0.34765	−1.343003376	0.71690218	0.7063	0.5024
Exercise at home^†^					
(Low-impact)	0.40091	−0.232001948	1.09509525	1.4932	0.2328
Exercise at home^†^					
(Moderate-impact)	−0.34016	−1.080813020	0.42538605	0.7117	0.3716
Exercise at home^†^					
(Without exercising)	0.21804	−0.396473331	0.89933723	1.2436	0.5063
Feeding habits care^‡^					
(No)	0.83978	0.193515638	1.44912535	2.3159	**0.0084**
Feeding habits care^‡^					
(A few times)	0.12702	−0.409418777	0.63382853	1.1354	0.6318
Feeding habits care^‡^					
(Yes)	−0.55852	−0.978639935	−0.14639141	0.5721	**0.0084**
COVID-19 diagnose					
(Yes, with laboratory tests)	0.52695	−0.475042890	1.36225307	1.6938	0.2529
COVID-19 diagnose					
(Yes, with symptoms)	1.38877	0.522492996	2.17601285	4.0099	**0.0009**

^§^Outdoor work was the reference category.

^†^High-impact was the reference category.

^‡^Sometimes was the reference category.

The bold values indicate the statistically significant results.

## 4. Discussion

This study aimed to investigate the effects of social confinement during the first wave of COVID-19 on certain living conditions of a group of volunteers participating in a cohort study of CDMX [previously described by Martínez-García et al. (29)]. Our results showed that during the first wave of COVID-19, the majority of the population that responded to our questionnaire belonged to low and medium social development strata, and the highest percentage of self-reported unemployment was among women. Men reported a higher prevalence of metabolic diseases and behavioral disorders than women. Social life and physical activity were the factors most affected during confinement, with a higher percentage of women reporting that they did not do any type of exercise at home and did not take adequate care of their diet.

Another finding of our study was that women reported a higher percentage of episodes of psychological (54.90%), verbal (36.27%), and emotional (31.37%) violence. Our results also identified different factors associated with violence, including age, unmarried status, neglect of feeding, and having presented symptoms related to COVID-19 without having undergone conclusive testing.

The COVID-19 pandemic has highlighted the need to promote self-care and healthy lifestyle habits to prevent chronic degenerative and metabolic comorbidities such as hypertension, diabetes mellitus, obesity, metabolic syndrome, and kidney disease (2). These comorbidities have been associated with the severity and worse prognosis of SARS-CoV-2 infection (31–33). In Mexico, as of March of 2022, Loza et al. reported the most prevalent comorbidities of near of six million COVID-19 confirmed cases related to hypertension (12.7%), obesity (10.5%), and diabetes (9.5%). In addition, the percentage of COVID-19-related deaths among people with diabetes and hypertension was 21.9 and 19.8%, respectively (2). Other studies have also reported that those who had died had presented with one or multiple comorbidities, nutritional deficiencies, and often had a history of smoking and a sedentary lifestyle, which could have made them more prone to serious complications (34, 35). Although the participants in our study came from a seemingly healthy population recruited well before the start of the pandemic, and at the time of applying the questionnaire, the majority neither had become ill with COVID-19 nor had their condition worsened. Our findings show that the participants already had a number of nutritional and metabolic comorbidities, as well as some mental and behavioral disorders, some of which were more prevalent in men than in women.

Exploring the effects of confinement on health habits such as eating disorders or physical activity is quite relevant in the context of a population such as the inhabitants of Mexico City, and given that in much of the Mexican population, there is a high level of food insecurity, a large problem of overweight and obesity, sedentary lifestyle, and high rates of the population with metabolic comorbidities (36–38). The preliminary reports of the study “PSY-COVID-19” (39), with more than 7,000 responses from Mexicans surveyed through a Google form, revealed that around half of the participants reduced their physical activity (more often men than women) and neglect their nutrition (more often women than men) during the first months of confinement (39). In our case, 38.31% of men and 42.78% of women did not perform any physical activity; however, 45.50% of men and 44.64% of women did take care of their nutrition. Derived from the experiences in terms of habit modification, the aforementioned study pointed out the importance of working on interventions to address situations such as appetite disorders or lack of motivation for physical activity related to adaptation to confinement and the ways of life of people (39).

On the other hand, although confinement helped to a great extent to contain the spread of COVID-19, the economic and social repercussions and the stress coping mechanisms that impacted health (e.g., excessive alcohol consumption and the use of cannabis, nicotine, and other drugs) are still being explored (40–42). In addition to impacting socioeconomic conditions, physical health, and mental health; social confinement also did so on family life and working conditions, with a greater effect on women (43). For instance, social confinement exposed gender inequalities related to the lack of employment and economic uncertainty (44). These vulnerable situations were perpetuated beyond the period of confinement and may have effects on the development of episodes of violence as those reported by the participants in this study (9.51% of prevalence of violence self-reported by women, see Table 4).

Consistent with these findings, in a recent Mexican study, based on data collected during 2020 through a remote survey of 47,819 women aged 15 years and older, Rivera et al. (25) reported an 11.5% of prevalence of violence against women, and the most reported acts were shouting, insults, or threats (4.3%) between 2020 and 2021 during the pandemic confinement. These authors also identified different factors associated with the episodes of violence, such as unemployment, being partially, and/or totally quarantined, being a family caregiver, binge drinking, and losing a family member to COVID-19 (25). The results from other studies regarding domestic violence during the same period of confinement in Mexico reported a 5.8% prevalence of episodes of violence against adult women, most of whom had already suffered some types of violence prior to the pandemic. This study revealed that the most reported acts of violence were emotional (4.3%), economical (2.1%), and physical (1.9%) (45). Unfortunately, our results also reflect this situation in some Mexican families, manifested mainly as psychological, verbal, and emotional violence, and their impact on socioeconomic vulnerabilities and mental health context has been little explored (44).

As already well-known, in situations of violence within homes, social isolation represents an opportunity to generate or maintain conditions of control and oppression, favored by the increase in contact time between the victim and the perpetrator, who is often the partner (27). Financial strain and isolation are also well-known domestic abuse risk factors, and both of these situations reduce the opportunities for people who are victims to ask for help (11). It is worth mentioning that the government of Mexico City made specific telephone lines available to the citizens for reporting gender violence and provided mental health support to those affected by the effects of confinement (23). According to Casas and Maldonado (14), there was a 45% increase in telephone complaints of domestic violence in Mexico during the second and third quarters of 2020. However, as Manrique de Lara and De Jesús Medina Arellano (44) pointed out, structural violence against women is often normalized in the Mexican context and has been exacerbated during the pandemic, affecting every sphere of society. Although health policies are being developed to provide life support services for victims of violence, the structural violence derived from the roles associated with care and domestic work that women face every day remains a neglected public health crisis in itself (44).

As we discussed, we found that suffering episodes of violence were significantly associated with being unmarried, as well as age, neglected feeding habits, or physical activity, and having had symptomatic COVID-19 infections. In connection to these issues, some studies have explored the effect of marital status (specifically, being unmarried) in relation to mental health during the lockdown from the beginning of the pandemic, but reports on the relationship with violence are somewhat limited. Ahmed et al. (46) found that women, students, unmarried individuals, and younger people were in more vulnerable positions in terms of demographics related to mental health during the pandemic in Bangladesh. Elhadi et al. (47) in turn, showed through multivariate analysis that being younger, women, unmarried, educated, or victims of domestic violence or abuse, having work suspension or increased workload, financial issues, suicidal thoughts, or a family member hospitalized due to COVID-19 were significantly associated with a high likelihood of mental disorders during the first months of the pandemic among the Libyan population. Additionally, Lee et al. (48) reported that higher levels of adverse mental health symptoms were associated with people who were single, reported a lower household income, had decreased support from friends or family, and increased stress at work or home during the COVID-19 pandemic in South Korea. Further studies may reveal how marital status may influence the observed effects of social confinement, particularly in the context of mental health. In fact, the medium- to long-term effects of social confinement on mental health are suspected to be substantial and remain far from being resolved (49).

The results of the present study further confirm some of these known trends and help contextualize them to highlight the interrelationship between biological, social, and emotional health conditions. In brief, this study has exposed some of the effects that social confinement during the COVID-19 pandemic had on certain living conditions, habits related to food, sleep, and physical activity, as well as people's daily lives and family relationships. This highlights the importance of interdisciplinary analysis, whose sole objective is to highlight the dimension of social vulnerabilities and their articulation with biological and mental factors for the generation of comprehensive health interventions.

The main living conditions modified among the volunteers were related to work, exercise, and food. With respect to violence and support networks, we consider them as a result of higher or structural categories. For example, different forms of violence can be related to historical–social processes that our society shares with the rest of Latin America, as well as being closely related to inequalities based on age, gender, and social capital (50, 51). Although some public policies have been implemented in Mexico to support victims of violence, it is largely unknown what effect they have had on the population during the pandemic (25). Within this study, we found that emotional and psychological conditions related to in-house violence were particularly salient. This highlights the importance of addressing mental health through public policies in our country in the post-pandemic era (52).

## 5. Conclusion

The findings of the present study revealed gender and socioeconomic differences in relation to the COVID-19 lockdown established in Mexico. These differences were observed in terms of places of work, the prevalence of metabolic diseases, mental, behavioral, and developmental disorders, as well as modified patterns of physical activity and social life.

The results of multivariate logistic models used to analyze the association between at-home violence episodes and various factors showed that such episodes were associated with *age, being unmarried, neglecting self-care (including eating habits)*, and *having suffered from COVID-19 infection with symptoms*. All of these factors suggest potential vulnerability. Although the prevalence of violence, in general, was similar between men and women, certain types of violence were significantly more prevalent among women, including economic, emotional, psychological, and verbal violence.

We have also noticed that although some public policy measures were implemented to support both the general and vulnerable populations during the lockdown, < 10% of the participants (with no statistically significant gender differences) reported receiving any support. This fact underscores the need to evaluate and redesign such support policies to maximize their social impact. Studies like this one can continue to provide evidence for the ongoing monitoring and improvement of social support policies and raise awareness of the often-overlooked living conditions of vulnerable populations.

## Data availability statement

The raw data supporting the conclusions of this article will be made available by the authors, without undue reservation.

## Ethics statement

The studies involving human participants were reviewed and approved by IRB of the National Institute of Cardiology Ignacio Chavez (protocol code 13-802). The patients/participants provided their written informed consent to participate in this study.

## Author contributions

SS-G performed the survey, organized the data, analyzed the data, and drafted the manuscript. AB-R performed the survey and organized the data. GG-E pre-processed and organized the data. EG-M supervised the survey and organized the data. MM-G devised and coordinated the project, contributed to the methodological strategy, performed calculations, analyzed the data, integrated the results, discussed results, drafted the manuscript, and co-supervised the project. EH-L designed the methodological approach, developed code, performed calculations, analyzed the data, discussed results, edited the manuscript, and co-supervised the project. All authors have read and approved the final manuscript.
